# pH Regulation of Electrogenic Sugar/H^+^ Symport in MFS Sugar Permeases

**DOI:** 10.1371/journal.pone.0156392

**Published:** 2016-05-26

**Authors:** Andre Bazzone, M. Gregor Madej, H. Ronald Kaback, Klaus Fendler

**Affiliations:** 1 Department of Biophysical Chemistry, Max-Planck-Institute of Biophysics, Frankfurt/M, Germany; 2 Departments of Physiology and Microbiology, Immunology, and Molecular Genetics, Molecular Biology Institute, University of California Los Angeles, Los Angeles, California, United States of America; University of Minho, PORTUGAL

## Abstract

Bacterial sugar symporters in the Major Facilitator Superfamily (MFS) use the H^+^ (and in a few cases Na^+^) electrochemical gradients to achieve active transport of sugar into the cell. Because a number of structures of MFS sugar symporters have been solved recently, molecular insight into the transport mechanism is possible from detailed functional analysis. We present here a comparative electrophysiological study of the lactose permease (LacY), the fucose permease (FucP) and the xylose permease (XylE), which reveals common mechanistic principles and differences. In all three symporters energetically downhill electrogenic sugar/H^+^ symport is observed. Comparison of the pH dependence of symport at symmetrical pH exhibits broad bell-shaped pH profiles extending over 3 to 6 pH units and a decrease at extremely alkaline pH ≥ 9.4 and at acidic to neutral pH = 4.6–7.5. The pH dependence can be described by an acidic to neutral apparent pK (pK_app_) and an alkaline pK_app_. Experimental evidence suggests that the alkaline pK_app_ is due to H^+^ depletion at the protonation site, while the acidic pK_app_ is due to inhibition of deprotonation. Since previous studies suggest that a single carboxyl group in LacY (Glu325) may be the only side chain directly involved in H^+^ translocation and a carboxyl side chain with similar properties has been identified in FucP (Asp46) and XylE (Asp27), the present results imply that the pK of this residue is switched during H^+^/sugar symport in all three symporters.

## Introduction

Sugar symporters in the large Major Facilitator Superfamily (MFS) [1,2] are found in all kingdoms of life [3]. The members usually contain of 12 transmembrane helices arranged in two pseudo-symmetrical 6 helix bundles surrounding a deep cavity that contains the substrate-binding site at the apex [4]. They are thought to function according to an alternating access mechanism [5] in which a single substrate-binding site is reciprocally accessible from the periplasmic (outward-facing state) or cytoplasmic sides (inward-facing state) of the membrane (see for recent reviews [6,7]). A common structural feature of MFS member, which suggests that the symporter members of the super family may have arisen by intragenic multiplication, is a repeat of four three-helix bundles organized as dual alternating inverted repeats in the two pseudo-symmetrical domains. Furthermore, substrate and H^+^ binding sites in distantly related symporters may be located in the same relative positions [8]. The structural organization also suggests that a common mechanistic pattern may be used for catalysis. However, a general, conceptual mechanism for the coupling of sugar transport to the H^+^ electrochemical gradient has been proposed for lactose/H^+^ symport only [9].

To date, crystallographic structures of a number of prokaryotic sugar/H^+^ symporters belonging to the MFS are available [10,11], but reliable functional assays of these symporters are scarce. In particular, conventional electrophysiological methods like patch- or voltage-clamping cannot be applied because prokaryotic membrane transport proteins frequently do not target to the plasma membrane of eukaryotic cells. Therefore, we have expressed, purified and reconstituted three different bacterial sugar symporters into proteoliposomes and subjected them to electrophysiological analyses using solid supported membrane (SSM)-based electrophysiology [12]. The time resolution and sensitivity of this method sheds new light on transport mechanisms and demonstrates that the three symporters studied have different properties beyond specificity for different substrates.

While differences between sugar symporters are interesting on their own, common features are also important because they reveal essentials of the mechanism. In this report we analyze and compare the pH dependence of the transport activity of the lactose/H^+^ (LacY), xylose/H^+^ (XylE) and fucose/H^+^ (FucP) symporters and draw conclusions regarding their symport mechanisms.

## Materials and Methods

### Plasmids and Construction of Mutants

Construction of the plasmids for pT7-5/WT LacY [13], pBAD-His A/WT FucP [14] and pET15b/WT XylE [15] have been described. Neutral substitution mutants for XylE and FucP were created by site-directed mutagenesis using the QuikChange Site-Directed Mutagenesis Kit (Stratagene).

LacY WT and mutant E325A were purified from *Escherichia coli (E*. *coli)* XL1-Blue cells (grown in LB media) transformed with pT7-5 plasmids harboring the appropriate *lacY* gene by using Co(II) affinity chromatography as described [13].

WT FucP, WT XylE and given mutants were purified from *E*. *coli* BL21(DE3). Cells were grown in 2YT media at 37°C, followed by induction at OD_600_ 0.8 with 0.2 mM IPTG (XylE) or 0.02% (w/v) arabinose (FucP), respectively, and growth was continued at 37°C for 3 h. After centrifugation (15 min, 4500*g* at 4°C) the cells were disrupted by a microfluidizer at 12.000 Psi followed by low speed centrifugation (15 min, 9500*g* at 4°C). The supernatant was used for ultracentrifugation (1 h, 100000*g* at 4°C) to harvest the membranes that were frozen and stored at -80°C.

Membranes were solubilized at 5 mg/ml total protein in 50 mM sodium phosphate, NaPi, (pH 7.5) containing 200 mM NaCl, 5 mM Imidazole, a protease inhibitor cocktail tablet (Complete Tablets EDTA-free EASYpack, Roche) and 1% (w/v) n-dodecyl-beta-D-maltoside (DDM) on ice. After centrifugation for 1 h (100000 g at 4°C), the supernatant was used for purification of the His-tagged proteins by metal affinity chromatography. After loading the sample and washing with 5 mM and 30 mM Imidazole in 50 mM NaPi at pH 7.5 and 0.01% (w/v) DDM, purified proteins were eluted with 200 mM imidazole in the same buffer and 0.01% (w/v) DDM.

The eluted sample (10 ml) was concentrated to 2–5 mg/ml, final concentration, by using a concentrator with a 10 kDa cut off (Amicon Ultra Centrifugal Filters, Ultracel-10K, Millipore). At the same time the buffer was exchanged with the buffer used for reconstitution (100 mM KPi, pH 7.5, 2 mM MgSO4). The final yield was about 2–4 mg of WT protein and 0.2–1 mg of mutant protein per liter culture. The concentrated sample was used directly for reconstitution. The purity of the protein samples was assessed by SDS/PAGE with either silver or coomassie staining.

### Reconstitution of Proteoliposomes

Reconstitution of purified proteins (2–20 mg/ml) was carried out with *E*. *coli* phospholipids (Avanti Polar Lipids, *E*.*coli* polar lipid extract). Preformed liposomes (0.2–2 ml, 10 mg/ml) dissolved in 1% (w/v) octyl glucoside (OG) and the protein suspension was mixed on ice to a concentration of 0.2 mg protein/mg lipid (LPR 5).

WT XylE, WT FucP and the mutant proteins were reconstituted using overnight incubation in 400 mg/ml BioBeads (SM-2 Adsorbent Media, BIO-RAD) at 4°C. After reconstitution the samples were diluted to 2.5 mg/ml lipid concentration, frozen in liquid nitrogen and stored at -80°C. For WT LacY and mutant proteins this procedure did not work. We, therefore, applied the previously established OG dilution method (1:100) [16].

### SSM-Based Electrophysiology

SSM measurements were performed as described [17,18]. After thawing the sample and sonication in a water bath (Sonorex RK 52 H, Bandelin) for about 30 s, 30 μl of proteoliposomes (2,5 mg/ml lipid at LPR 5) was allowed to adsorb for 1–2 h to an octadecanethiol/phosphatidylcholine hybrid bilayer on a gold surface (the sensor).

Two different solution exchange protocols were used. 1) For the measurements under symmetrical pH conditions a single solution exchange configuration [18] was employed that consisted in 3 phases of 0.5 s duration each: Flow of nonactivating solution (NA), activating solution (A), and nonactivating solution (NA). Only the activating solution contained the sugar. 2) For the measurements under asymmetrical pH conditions a double solution exchange configuration [18] was employed where an additional resting solution (R) phase of 2 s was added to the end of the protocol. This allowed the incubation of the sample at a different pH: An incubation time of 3 to 20 min depending on the pH change adjusts the inner pH of the proteoliposomes to pH of R and establishes a pH gradient at the beginning of each measurement [18].

Instruments with different time resolution were used as required. The high time resolution set up with a valveless diverted fluidic geometry had a flow rate of about 0.5 ml/s and a time resolution of 5 ms. The low time resolution set up had a flowrate of 2 ml/s and a time resolution of 15 ms [19].

Currents were recorded throughout the entire time and amplified with a current amplifier set to a gain of 10^9^ V/A and a rise time of 10 ms (low time resolution set up) and 3 ms (high time resolution set up).

All solutions were buffered in 100 mM KPi at a given pH value. The buffers used with LacY contained 1 mM dithiothreitol (DTT) in addition. The activating solution contained the respective sugar at a given concentration to induce the symport reaction.

The activity of the transporter was tested at neutral pH before and after each measurement to ensure that there was no activity loss during the measurement. Therefore, the proteins are stable for the duration of the electrophysiological measurement at the extremes of pH used here.

For the monensin control measurements 10 μM monensin was added to all solutions, A, NA and R. For equilibration the sample was incubated in monensin containing R solution 20 minutes prior to the measurements.

### Data Analysis and Representation

When different transient currents are shown in one graph, all traces were recorded from the same sensor and the transients shown are representative results. Similar transients were observed with at least three different sensors. The peak currents from each sensor were normalized to the respective maximum current, followed by averaging.

The crystallographic coordinates of XylE structure models with the symporter in the substrate-free state (pdb-id: 4qiq) [15] or with bound xylose (pdb-id: 4gby) [20] were analyzed using UCSF Chimera software [21].

### Experimental Determination of K_m_ and pK_app_ Values

For determination of K_m_ values the concentration dependence of the normalized peak currents (I_norm_) was fitted with a hyperbolic equation (*c* = sugar concentration):

$${\text{I}}_{\text{norm}}={\text{I}}_{\text{max}}\frac{\text{c}}{\text{c}+{\text{K}}_{\text{m}}}$$

For determination of the apparent pK values, pK_app_, the pH dependencies of the normalized peak currents were fitted using titration functions. These model functions are functions of a single H^+^ binding group with pK_1app_ or pK_2app_ or two H^+^ binding groups with pK’_2app_ and pK”_2app_:

Acidic inactivation:

$${\text{I}}_{\text{norm}}\left(\text{pH}\right)=\frac{{\text{I}}_{\text{max}}}{1+10^{{\text{pK}}_{1\text{app}}-\text{pH}}}$$

Alkaline inactivation:

$${\text{I}}_{\text{norm}}\left(\text{pH}\right)=\frac{{\text{I}}_{\text{max}}}{1+10^{\text{pH}-{\text{pK}}_{2\text{app}}}}$$

Biphasic alkaline inactivation:

$${\text{I}}_{\text{norm}}\left(\text{pH}\right)=\frac{{\text{I}}^{\prime }}{1+10^{\text{pH}-{\text{pK}}_{2\text{app}}^{\text{'}}}}+\frac{{\text{I}}^{\prime \prime }}{1+10^{\text{pH}-{\text{pK}}_{2\text{app}}^{\text{''}}}}$$

I’ and I” are the relative maximal contributions of the two phases with pK’_2app_ and pK”_2app_.

pH dependent sugar binding was analyzed using sequential H^+^/sugar equilibrium binding. C is the symporter with the respective pK of H^+^ and K_D_ of sugar binding:

$$\text{C}\overset{\text{pK}}{\Leftrightarrow }\text{CH}\overset{{\text{K}}_{\text{D}}}{\Leftrightarrow }\text{CHS}$$

Assuming equilibrium substrate binding the pH dependent K_m_ for sugar binding K_m_(pH) is given by:

$${\text{K}}_{\text{m}}\left(\text{pH}\right)={\text{K}}_{\text{D}}\left(1+10^{\left(\text{pH}-\text{pK}\right)}\right).$$

## Results

### Sugar Concentration Dependence

Proteoliposomes containing one of the reconstituted sugar symporters LacY, FucP or XylE were characterized by SSM-based electrophysiology. Sugar concentration jumps initiated sugar/H^+^ symport, and charge transfer was measured by capacitive coupling. Therefore only transient currents were recorded. The peak current of the transient represents a good approximation of the current generated by the steady-state activity of the symporter [18].

**Fig 1:**
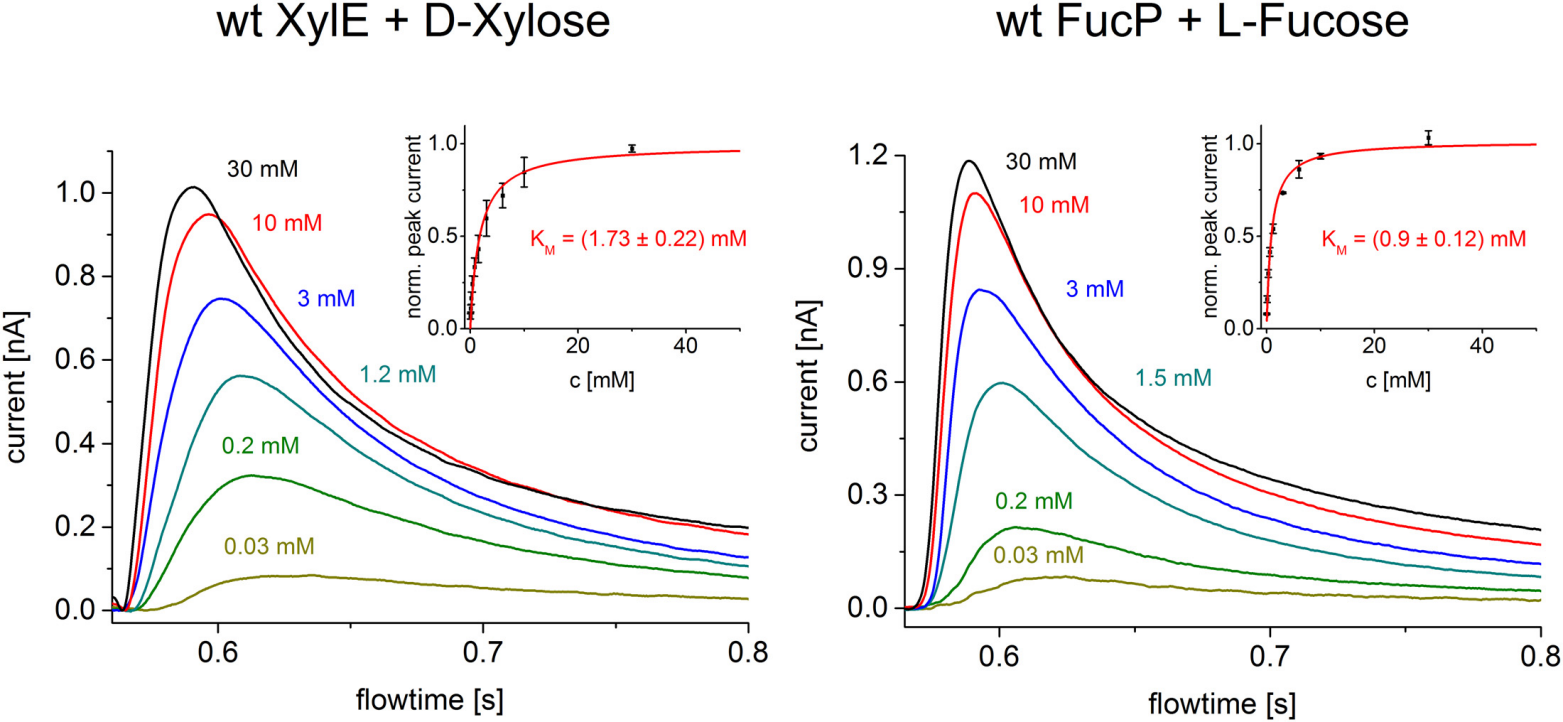
Sugar concentration dependence. Transient currents obtained with XylE and FucP proteoliposomes after a sugar concentration jump at pH 7.6 using the low time resolution set up. The different concentrations of D-Xylose and L-Fucose are indicated. The inset shows mean and standard deviation of the normalized peak currents obtained from three different sensors for all sugar concentrations used. The K_m_ was determined from a hyperbolic fit.

**Fig 2:**
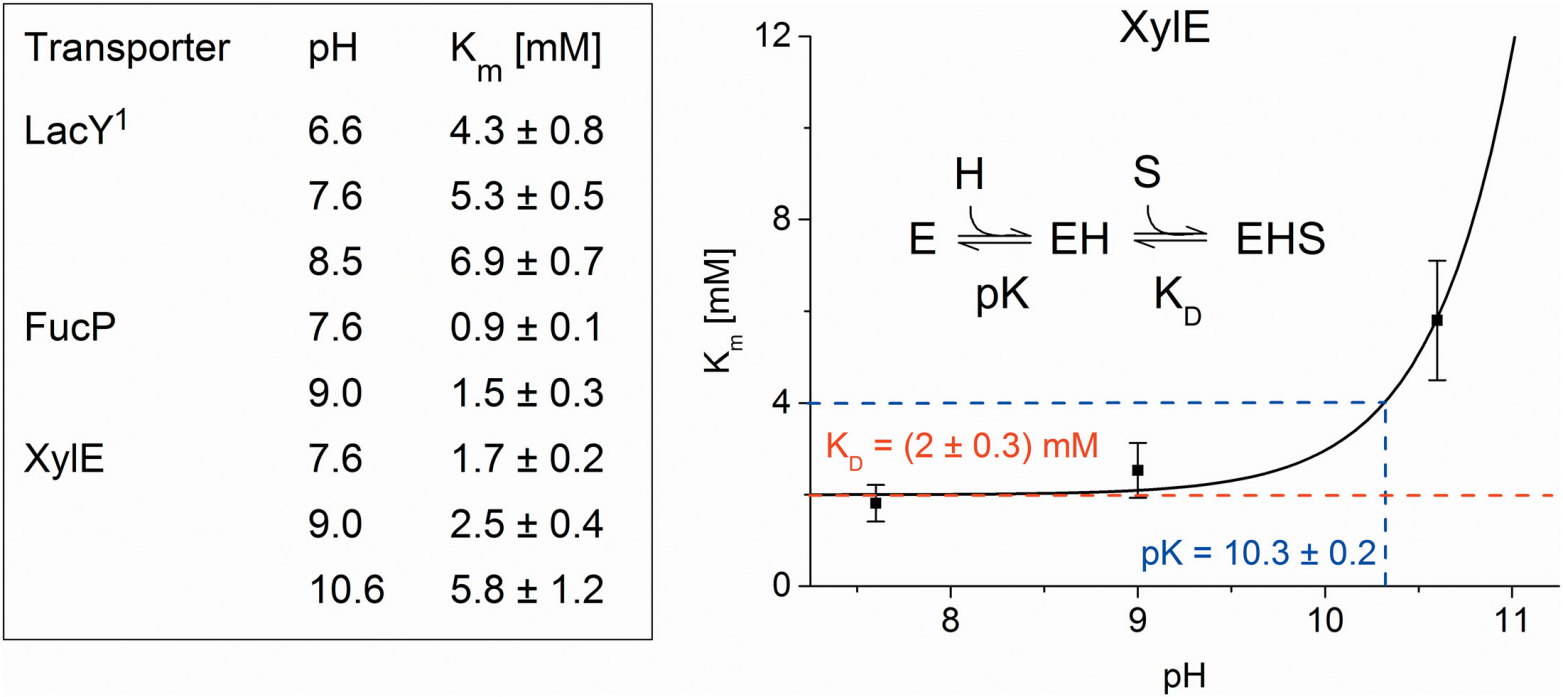
pH dependence of K_m_ values. Table: K_m_ values at different pH for the main substrates α-D-Lactose, L-Fucose and D-Xylose of LacY, FucP and XylE, respectively determined from a hyperbolic fit to the peak currents as shown in Fig 1. ^1^Data from [31]. Graph: pH dependence of K_m_ for XylE. The line is a fit to the data using a model function for sequential H^+^—sugar binding as indicated in the figure.

K_m_ values for sugar transport were obtained by measuring the peak currents at different sugar concentrations (Fig 1). K_m_ values are in the range of a few mM (Fig 2) and show no significant variation with pH in the physiological range but increase at very high pH values, as observed for XylE (Fig 2). This is consistent with the pK_app_ of ~10.5 observed for galactoside binding to LacY [22], as well as the interpretation that protonation precedes sugar binding over the physiological range of pH [23]. A fit to the XylE data using a model function for sequential H^+^/sugar binding (see Materials and Methods) yields a K_m_ = 2 mM for xylose and a pK_app_ = 10.3 ± 0.2 (Fig 2).

### Effect of pH

**Fig 3:**
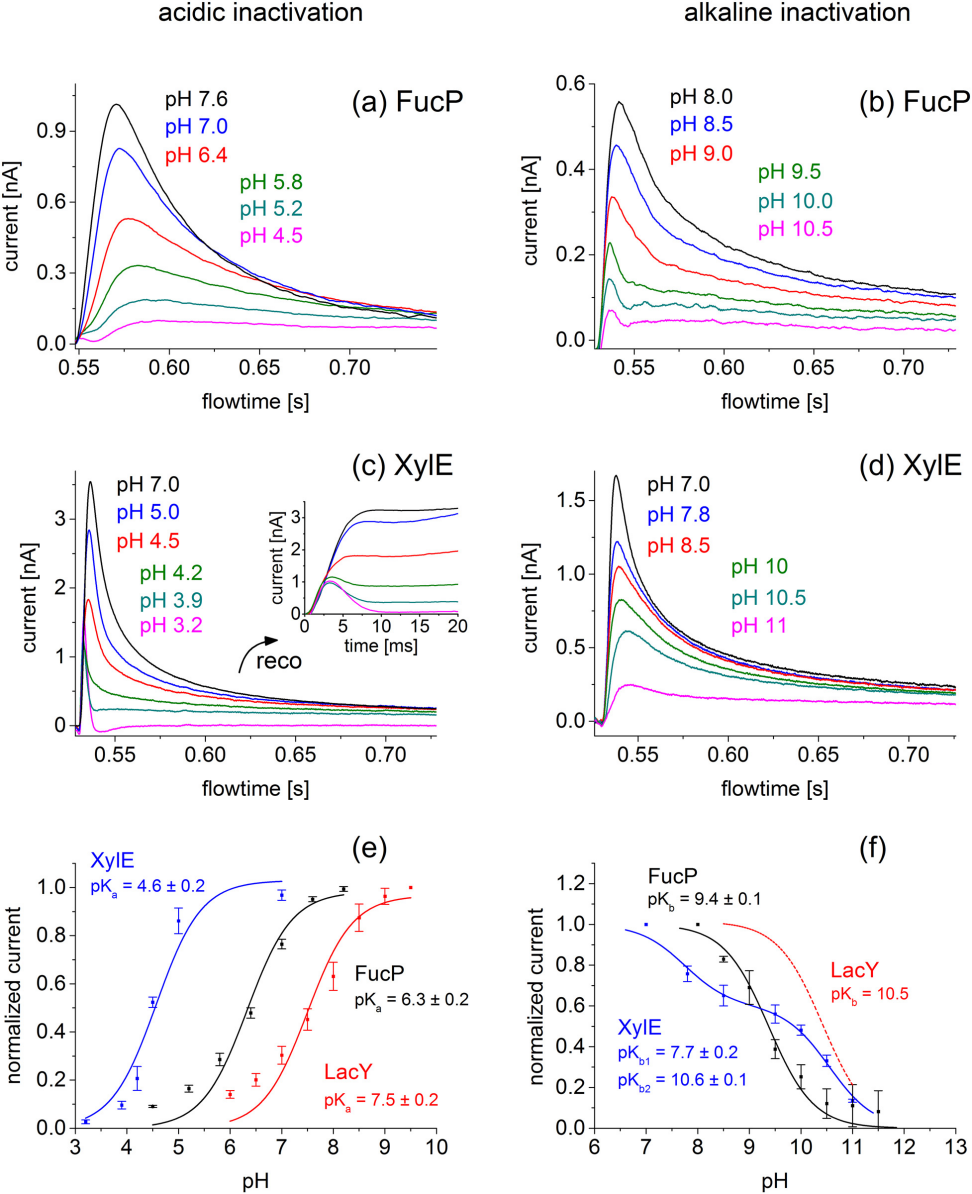
pH dependence of peak currents for LacY, FucP and XylE in the acidic (left) and basic (right) pH range. (a, b) Transient currents obtained with FucP proteoliposomes after a 30 mM L-Fucose concentration jump in the acidic pH range using the low time resolutions set up (a) and in the basic pH range using the high time resolution set up (b). (c, d) Transient currents obtained with XylE proteoliposomes after a 30 mM D-Xylose concentration jump in the acidic pH range (c) and the basic pH range (d) using the high time resolution set up. The inset of (c) shows reconstruction of the symporter currents using circuit analysis (see Text). Note that the different peak currents for the same pH values in (a) and (b) as well as (c) and (d) is a result of different amounts of proteoliposomes adsorbed to the sensor. Acidic and alakaline inactivatation was measured on different sensors.(e,f) Peak currents and determination of pK values for LacY (red), FucP (black) and XylE (blue) proteoliposomes after a sugar concentration jump of 100 mM alpha-D-Lactose, 30 mM L-Fucose and 30 mM D-Xylose, respectively. For comparison transient currents for LacY were determined using the low time resolution set up (data not shown) and average peak currents are included. The solid line is a fit to the data using a titration function as described in Materials and Methods. Experimental conditions as described in panel e. The deviation of the data points from the fitting curve under extreme pH conditions for LacY and FucP is possibly due to the increasing influence of presteady state components.

For all symporters, the shape and magnitude of the transient currents depend strongly on pH, and characteristic differences are observed (Fig 3). The peak currents of FucP and XylE decrease at acidic and alkaline pH (Fig 3a–3f). However, the currents recorded for XylE in the acidic to neutral pH range show a distinct behavior not observed for FucP. Between pH 4.5 and 4.2 a distinctly rapid decay in the time course of the currents with XylE is observed. This is indicative of a rapid pre steady-state charge displacement in the symport current. Such behavior was reported for LacY previously, which was resolved at acidic pH because steady-state turnover is inhibited by the high H^+^ concentration [24]. To discriminate between steady-state and pre steady-state charge translocation the underlying symport currents were reconstructed using the electrical properties of the SSM [18] and are shown in the inset of Fig 3c. At acidic pH, only a rapid charge displacement is observed in the reconstructed currents, which is gradually superimposed by steady-state transport activity of XylE as pH rises. Since our interest concerns the steady-state activity, the acidic pH profile of XylE (Fig 3e) was generated using steady-state values of the reconstructed currents.

Fig 3e and 3f shows the pH profile using normalized currents. For comparison, we include a pH dependence of LacY transport activity. As a consequence of LacY protein stability, activity could not be determined above pH 11 where LacY becomes denatured. However, a titration function with a pK_app_ ≃ 10.5 is included for LacY as a benchmark [22].

All symporters are inactive at extreme acidic and alkaline pH with a plateau of ~ 3–6 pH units. Note that the pH profiles are shifted to alkaline pH in the order XylE, FucP, LacY. To characterize the pH profiles, a titration function was fitted to the data (see materials and methods) and the pK_app_ values were determined, which define the onset of activity in the acidic (pK_1app_) and the decrease in the alkaline pH range (pK_2app_) (Table 1).

**Table 1:** Apparent pK values for acidic and alkaline inactivation.

	putative H^+^ binding residue	H^+^ release: pK_1app_	H^+^ binding: pK_2app_		average current at optimal pH
**LacY**	**E325**	7.5 ± 0.2	~10.5**		2 ± 0.5 nA
**XylE**	**D27**	4.6 ± 0.2	(7.7 ± 0.2)*	10.6 ± 0.1	2.4 ± 0.7 nA
**FucP**	**D46**	6.3 ± 0.2	9.4 ± 0.1		0.9 ± 0.4 nA

Apparent pK values for acidic inactivation pK_1app_ and alkaline inactivation pK_2app_ were determined by least square fitting to the data in Fig 3e and 3f. For comparison the average current amplitudes at optimal conditions of the electrophysiological measurements are given. The table also summarizes the putative H^+^ binding residues of the sugar/H^+^ symporters.

*As explained in the text the pK_2app_ = 7.7 is not relevant for sugar/H^+^ symport.

** data from [22].

A feature of the XylE transport activity in the neutral to alkaline pH range is the biphasic decrease, which is characterized by two different apparent pK_app_ values of 7.7 and 10.6 (Table 1). To clarify the significance of the two pK_app_ values, the pH dependence of the K_m_ for D-xylose was determined at neutral and extremely alkaline pH (Fig 2). Accordingly, a pK_app_ = 10.3 ± 0.2 was determined suggesting that the pK_app_ for sugar binding and consequently for sugar/H^+^ symport is likely 10.6 ± 0.1. The apparent pK_app_ of 7.7 probably represents a modulation of electrogenicity or turnover involving a H^+^ binding residue other than that relevant for transport.

### Kinetic Isotope Effect of Deuterium Oxide

With LacY, lactose efflux in the absence of the H^+^ electrochemical gradient is inhibited 3- to 4-fold by D_2_O over the neutral pH/pD range [16] indicating that H^+^ transport is rate-limiting for this reaction, and this was confirmed by SSM based electrophysiology recently [25]. Moreover the kinetic isotope effect is not observed for the pre steady-state current of E325A LacY [25]. In this mutant, the carboxyl side chain at position 325 is substituted with a neutral amino acid and no H^+^ release occurs.

**Fig 4:**
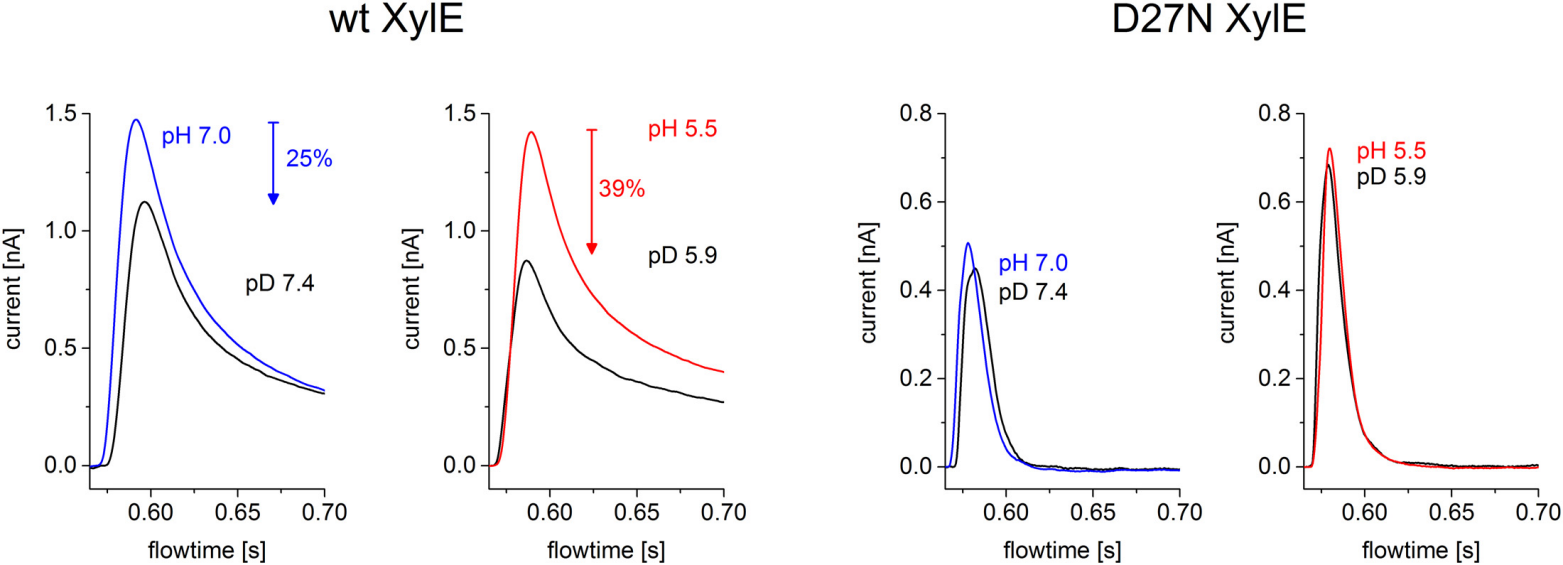
kinetic isotope effect of wildtype XylE and D27N XylE. For both samples two different pH values were compared (5.5 and 7.0) and 10 mM of D-Xylose was used as the activating compound. The solutions were prepared in 99% D_2_O and 100% H_2_O respectively. After switching between H_2_O based and D_2_O based solutions the sensor was incubated for 5 min. The low time resolution set up was used.

Similar experiments were performed for WT XylE and D27N XylE to test whether H^+^ transport is also limiting for lactose-driven H^+^ influx with XylE (Fig 4). For WT XylE at pH 7.0 and pH 5.5 the peak current decreased in D_2_O solutions by 25% and 39%, respectively, indicating that H^+^ transport is rate-limiting. This effect is larger at pH 5.5 because of proximity to the pK_app_ of 4.6 for acidic inactivation of XylE. The observations clearly agree with those presented for WT LacY [16].

For D27N XylE, which has properties similar to E325A in LacY [26], no significant D_2_O-related amplitude change in the peak current is observed. Overall the results obtained with XylE are similar to those observed with LacY: a reduction of the currents in the wild type symporter and no effect of D_2_O in the transport deficient mutant. This is strong evidence that in XylE, similar to LacY, deprotonation is also rate limiting for sugar-driven H^+^ transport in the proximity of pK_app_ for acidic inactivation.

### Transport Activity at Asymmetrical pH Conditions

**Fig 5:**
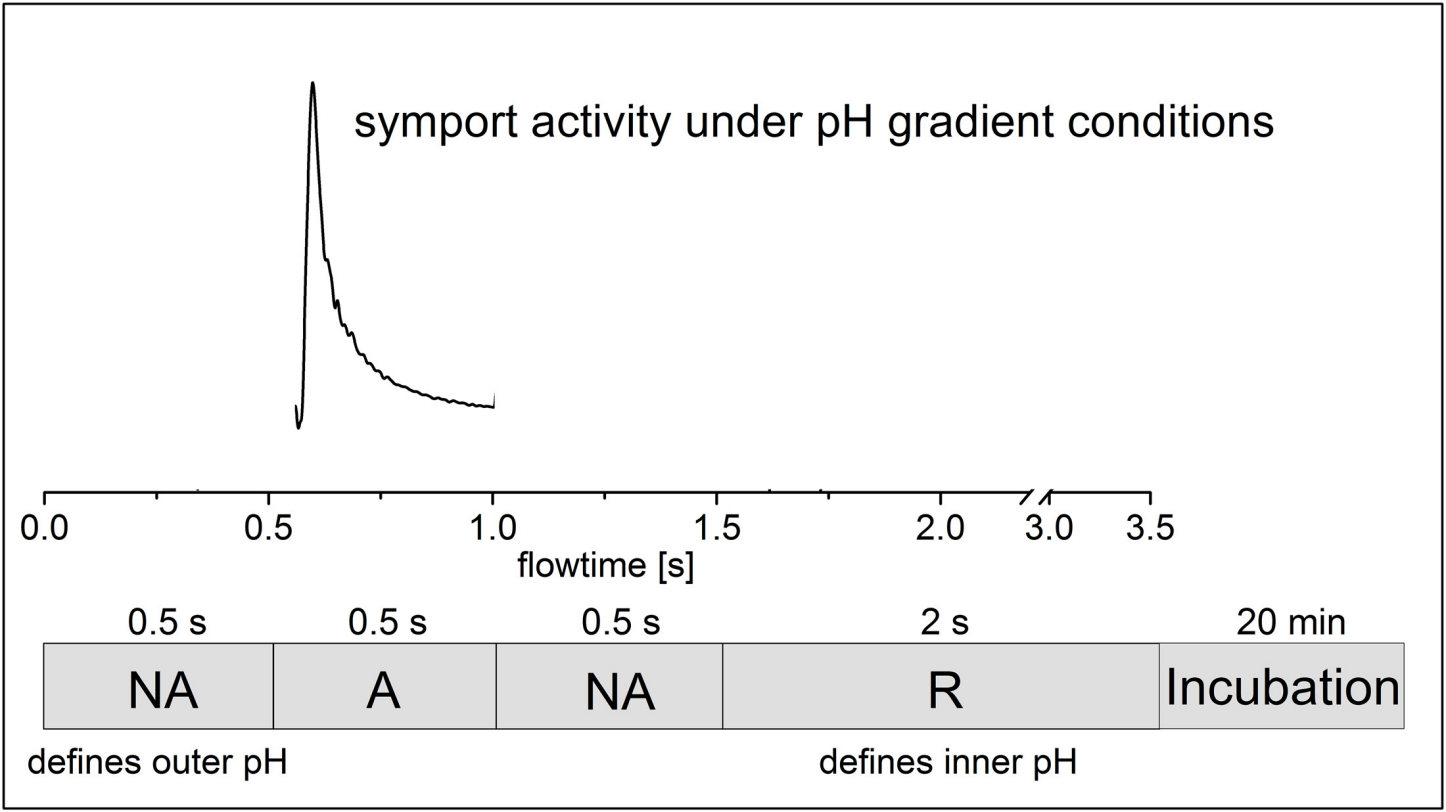
Double solution exchange flow protocol used for the electrophysiological measurements under asymmetrical pH conditions. The bar at the bottom shows the sequence of solution flow. At the end of each experiment the resting solution (R) flows and exchanges the outer pH to pH(R). The following incubation time (3 to 20 minutes depending on the gradient) equilibrates the inner pH of the proteoliposomes to pH(R). At the beginning of each experiment the flow of the nonactivating solution (NA) with pH(NA) then creates a pH gradient. After 500 ms the activating solution (A) with the same pH provides the sugar and initiates symport activity under pH gradient conditions. The resulting transient current is recorded. Since the pH gradient dissipates rapidly the absolute pH values inside the proteoliposomes should be taken as approximative.

From the pH profile of the transport activity we suggest that the two different pK_app_ values (Table 1) correlate with H^+^ binding (pK_2app_) and H^+^ release (pK_1app_) of the symporters. Since the two processes take place at opposite sides of the membrane, transport was measured under asymmetrical pH conditions, i.e. pH inside and outside the proteoliposomes was different. For example, with sugar-driven H^+^ influx, acidic inactivation should be influenced only by internal pH (low pH inhibits H^+^ release), while alkaline inactivation should be a function of external pH only (high pH inhibits H^+^ binding). Using a double solution exchange protocol, a pH gradient can be established [18] and symporter currents at asymmetrical pH can be recorded after a subsequent sugar concentration jump (see Fig 5 and Materials and Methods).

The pH values of the solutions were chosen to achieve a maximum activity change for each symporter in the respective pH profile (Fig 3e and 3f). Specifically, the pH of one solution was close to the optimal pH, whereas the pH of the other solution was 3.6 pH units higher for alkaline inactivation and 3.6 pH units lower for acidic inactivation, respectively. This corresponds to an expected activity drop of more than one order of magnitude. Acidic inactivation was investigated for all 3 symporters, and alkaline inhibition was analyzed for FucP and XylE only because LacY is unstable at extreme alkalinity. The mechanism of alkaline inhibition of XylE is complex and differs from that of the other symporters (Fig 3f).

**Fig 6:**
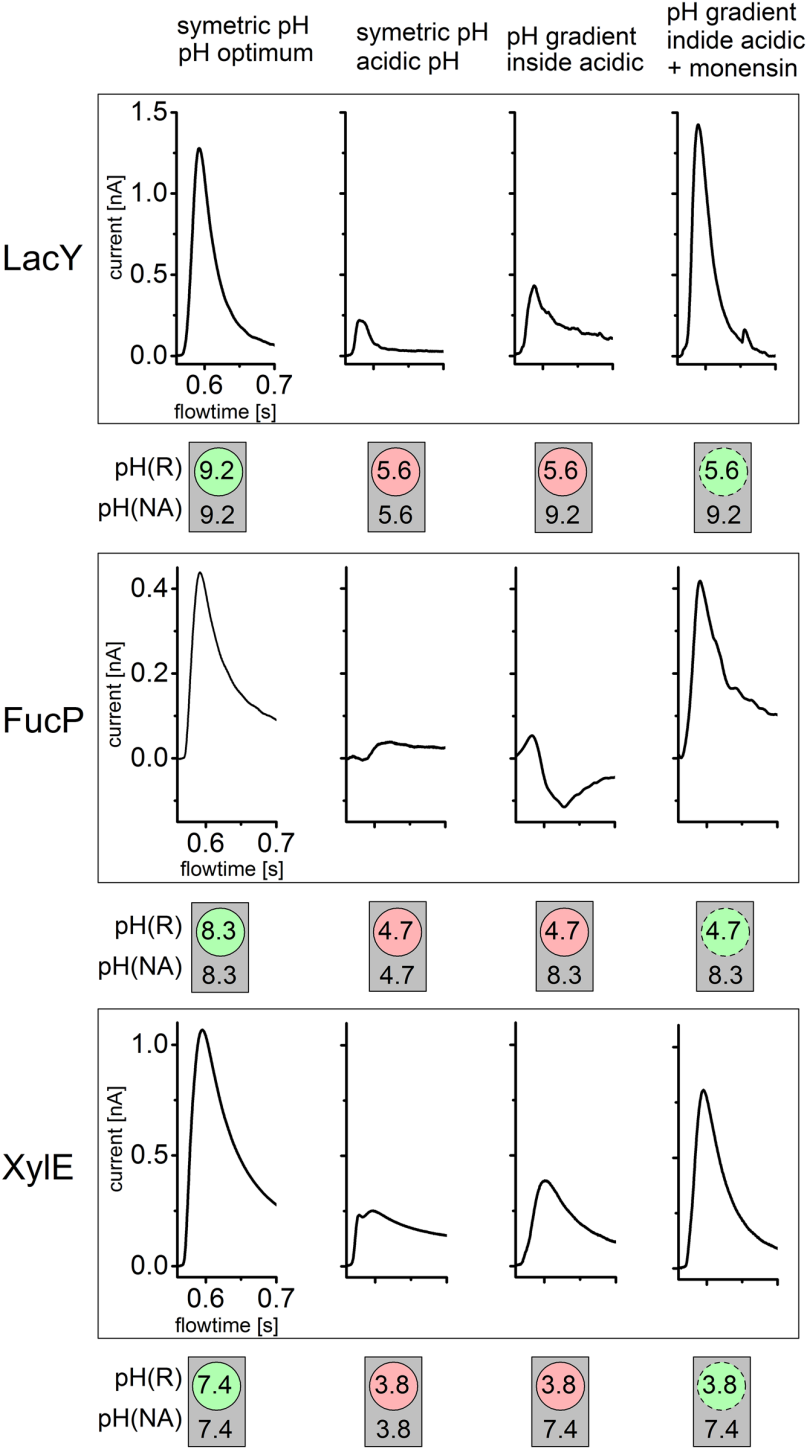
Acidic inactivation of LacY, FucP and XylE is due to the pH at the proton release site. The transient currents under symmetrical and asymmetrical pH conditions for LacY, FucP and XylE before and after addition of monensin are shown. The pH values were chosen so that inactivation of symporter activity at acidic pH values could be observed (compare Fig 3e). The pH values for the resting solution (R) and the nonactivating as well as activating solutions (NA and A) are indicated inside circles symbolizing the proteoliposomes and outside these circles respectively. The dashed circles symbolize leaky proteoliposomes due to monensin addition. There the pH gradient rapidly dissipates during the first flow of nonactivating solution (compare Fig 5) and the intraliposomal pH adjusts to the outer pH. For all symporters a constant pH gradient |ΔpH| = 3.6 was chosen. All solutions were prepared as described in Materials and Methods and Fig 3. The peak currents representing turnover rates are influenced mainly by the inner pH where proton release takes place and are independent of the outer pH where proton binding occurs. This is represented by the green (inside activating pH) and red (inside inactivating pH) circular areas. In contrast the grey square indicates that activity is independent of the pH of the outer solution.

Analysis of acidic inactivation is shown in Fig 6. The pH inside and outside of the proteoliposomes after the pH jump is schematically depicted at the bottom of each panel. These are idealized values corresponding to the pH of solution R (inside) and A and NA (outside), respectively. Although the pH inside the proteoliposomes varies with time due to passive H^+^ influx, the experiments show that a pH gradient is present for sufficient time to measure the sugar-induced transient currents.

The first two transient currents are recorded at symmetrical pH using the single solution exchange protocol described for previous measurements. The following two traces result from a double solution exchange protocol generating a pH gradient across the liposomal membrane. As control, the experiments were repeated in presence of the K^+^/Na^+^/H^+^ exchanger monensin, which allows rapid electroneutral dissipation of the pH gradient. Under these conditions the internal pH would be approximately equal to the external pH at the time of the transport measurement.

For all symporters the same behavior is observed: acidic pH inside yields small signals comparable to that recorded at symmetrical acidic pH regardless of external pH. This suggests that internal pH is responsible for acidic inactivation, and because transport is driven by an inwardly-directed sugar gradient, this is the pH at the H^**+**^ release site.

**Fig 7:**
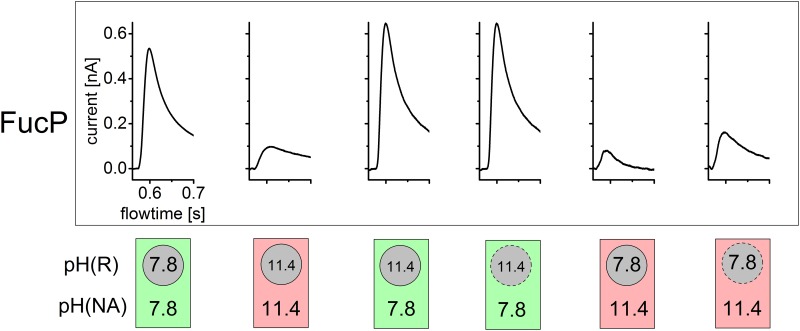
Basic inactivation of FucP is due to the pH at the proton binding site. The transient currents under symmetrical and asymmetrical pH conditions for FucP before and after addition of monensin are shown. The pH values were chosen so that inactivation of symporter activity at basic pH values could be observed (compare Fig 3f). Further details and all conditions as described in Fig 6. The turnover rates for these pH values are influenced only by the outer pH where proton binding takes place and are independent of the inner pH where proton release occurs. This is represented by the green (outside activating pH) and red (outside inactivating pH) squares. In contrast the grey circular areas indicate that activity is independent of the pH of the inner solution.

Alkaline inactivation of FucP is shown in Fig 7 where alkaline pH inside is shown to yield large signals comparable to that recorded at neutral pH. This indicates that the internal alkalinity enhances deprotonation. Consistently, alkaline pH externally suppresses the signal. Therefore, the pH at the H^**+**^ uptake side is responsible for alkaline inactivation.

As internal control we repeated acidic inactivation experiments (Fig 6) using increasing incubation times of the first NA solution flow period (Fig 5). Increasing the NA solution incubation time leads to dissipation of the pH gradient and adjustment of internal pH to external pH. Indeed, sufficiently long incubation times restored the transient current to its initial value at symmetrical pH (data not shown), which, like the monensin experiments, confirms internal pH as the source of inactivation.

## Discussion

A detailed molecular understanding of the mechanism employed by the sugar symporters requires structure information and detailed functional analysis. To date, the crystallographic structures of five H^+^-coupled MFS sugar symporters have been solved at reasonably high resolution: LacY [4,27,28], FucP [14], XylE [15,20], GlcP [29] and MelB [30]. Detailed functional information is only available for LacY and MelB, arguably the best studied sugar symporters. We now present complimentary electrophysiological information about FucP and XylE to reveal common concepts and differences in sugar/H^+^ symport by these proteins.

For LacY, it has been proposed that high apparent pK during substrate binding and low apparent pK during substrate release is a characteristic property of the transport process [9]. This is now shown to be a general property of at least three different H^+^ coupled MFS sugar symporters. In this report, the pH profiles of the transport activities of LacY, XylE and FucP are compared and a direct assignment of the high apparent pK to substrate uptake and the low apparent pK to substrate release in the symporter is made. A comparison of experimental data (see Table 1) reveals that a general feature of the pH profiles is an activity range of ~ 3–6 pH units. If a simple alternating access mechanism is assumed with a single H^+^ binding site, the broad activity range implies that the pK of this site is switched during the transport reaction from a high to a low pK.

### Electrophysiological Characterization of XylE and FucP

As with LacY [31], sugar gradient driven positive transient currents are recorded for XylE and FucP showing the characteristics of steady-state translocation of positive charge into the proteoliposomes (Fig 1). The concentration dependence of the respective main substrates D-xylose and L-fucose saturates with K_m_ values of 1–2 mM (Figs 1 and 2), comparable to previously published K_D_ values obtained from ITC measurements: K_D_(FucP) = 0.47 ± 0.02 mM [14]; K_D_(XylE) = 0.35 ± 0.03 mM [20]. In all cases, K_m_ increases with pH indicating that H^+^ is bound before the sugar as proposed for LacY [6].

### H^+^ Uptake and Release during Sugar Transport

The pH dependence of sugar transport gives insight into the role of H^+^ during the sugar/H^+^ symport process. All three symporters are inhibited at extreme acidic and alkaline pH (Fig 3) with an activity plateau in between. Inhibition is characterized by specific apparent pK values summarized in Table 1. The values for acidic inhibition of the symporters differ by ~ 3 pH units from pK_1app_ = 4.6 to 7.5. Alkaline inhibition described by pK_2app_ occurs at very high pH ≥ 9.4. This yields a remarkably broad pH range of ~ 6 pH units for XylE and of ~ 3 pH units for LacY and FucP sugar/ H^+^ symport.

What is the mechanistic background for inhibition of sugar transport at acidic and alkaline pH? Importantly, the electrophysiological assays in Fig 3 show sugar gradient driven activity at symmetrical pH. However, by applying conditions generating a pH gradient (Figs 6 and 7), acidic inhibition can be attributed to internal pH while alkaline inhibition is found to be due to the external pH. This allows the experimental estimation of the pK for internal H^+^ release (pK_1app_) and the pK for external H^+^ binding (pK_2app_).

Electrophysiological analysis agrees very well with the functional data available for LacY, where a pK of ~ 10.5, was determined from pH dependent sugar-binding studies [22]. Also, it was demonstrated that in the absence of a H^**+**^ electrochemical gradient (as is the case with the electrophysiological experiments of Fig 3), the rate-limiting process is the release of H^+^ to the inside [9] with a pK_app_ of ~7.5 [24,31]. From these data an activity profile can be derived with inhibition at acidic pH where H^+^ release becomes rate limiting and inhibition at alkaline pH where H^+^ binding limits transport activity. Indeed, this is observed for all three symporters (Fig 3). Further support comes from the deuterium kinetic isotope effect with WT XylE (Fig 4), which indicates that H^+^ release is rate-limiting in XylE at acidic pH.

What is the physiological purpose of a range of 3–6 pH units in the activity profiles? Enterobacteria colonizing the lower human intestine face an environmental pH of 6.6 to 7.3 [32] and maintain their cytoplasm at ~ pH 7.5. Therefore, the large variation of uptake and release pKs between symporters was a surprise, given that they have all evolved to take up sugars at comparable environmental conditions. Clearly, transport activity is high for all symporters in the physiological pH range. The low apparent H^+^ release pK_app_ of XylE of 4.6 is probably of no physiological significance because cytoplasmic pH is constant at 7.5. However, the high apparent binding pKs (≥ 9.4, e.g. in FucP) may be important for survival of the organism at extreme environmental conditions.

### Symporter Symmetry and Orientation in the Proteoliposomes

It has been suggested that sugar symporters may be functionally symmetrical [9]. Studies with LacY in the absence of a H^**+**^ electrochemical gradient have shown that galactoside affinity is essentially identical on both sides of the symporter [33], and the apparent pK values for influx (pK 7.5, [24]) and efflux (pK 8.5, [16]) are similar as well as the rates of influx and efflux [23]. Therefore, with LacY, the pK asymmetry observed in the present and previous studies (pK_1app_ ≠ pK_2app_) may be a feature of the transport mechanism rather than one of the two conformations of the protein. In other words, the direction of sugar transport determines pK_app_ and the release pK_app_ is always much smaller than the uptake pK_app_ regardless of whether sugar and H^+^ are transported into or out of the cell or liposome [9].

For XylE and FucP experimental data about functional symmetry is not available and the orientation of these symporters in the proteoliposomes is unknown. LacY is > 85% in a right-side-out orientation in proteoliposomes [34]. Since XylE and FucP have been reconstituted using similar methods as with LacY, it seems likely that they exhibit the same orientation. In addition, no evidence for two populations with different orientations was found in our functional analysis. Model calculations based on a asymmetrical 6-state kinetic model with different pKs of the outside and inside oriented conformations showed that in a mixed orientation biphasic inactivation is expected on the acidic side of the pH profile (see S1 File). However, in all cases monophasic acidic inactivation of LacY, XylE and FucP is experimentally observed. In addition, only when the high pK binding site is facing outside, the distinctive broad pH profiles as found experimentally are obtained from the model calculation. Thus, like LacY, XylE and FucP may likely be predominantly oriented in a single orientation (most probably right-side-out) in the proteoliposomes.

However, if the symporters are functionally highly symmetrical as discussed above for LacY, orientation is not an issue. In this scenario, single titration curves would be observed in samples with mixed orientation and apparent pK values for H^+^ binding and release could be determined regardless of symporter orientation. In both cases our analysis is correct, but the pK values have to be interpreted differently. For symmetrical symporters, the pK values are associated with H^+^ binding and release independent of transport direction. In an oriented sample with asymmetric symporters, the pK values are associated with the respective side (extracellular or cytoplasmic) of the symporter.

### Mechanistic Implications

As shown previously for LacY [31], electrophysiological analysis of XylE and FucP is consistent with a sugar/H^+^ symport mechanism. Transport activities in the sugar-driven H^+^ symport mode estimated from the transient currents are similar for LacY and XylE and ~ two times smaller for FucP (Table 1).

Interestingly all symporters have an extremely alkaline pK_app_ > 9.5 for H^+^ uptake. This raises the question how the H^+^ can later be released. Assuming a typical second order binding rate constant for a diffusion controlled reaction of ~ 10^10^ M^-1^s^-1^, this yields a H^+^ release rate of ~ 3 s^-1^, a value at least an order of magnitude too low for effective turnover. Therefore the symporter must have a means of lowering the pK during its reaction cycle. Indeed we find release pK_app_s between 7.5 and 4.6. Although these apparent pKs do not necessarily represent the pK of the binding site, the considerations suggest that a pK switch is present in MFS sugar symporters. A possible solution is a pK switch between the periplasmically oriented and the cytoplasmically oriented conformations of the symporter. However, this renders the symporter asymmetrical, which seems to be in contradiction with the experimental data from LacY.

How could such a pK-switch be realized? In LacY, the switching of the pK has been suggested to originate from a positively charged residue, Arg302, approaching the H^+^ binding site (Glu325), thereby lowering the pK by 3 pH units upon dissociation of galactoside and reorientation of the substrate binding site from out to inside [9]. Carboxylates analogous to Glu325 in LacY have been identified in XylE (Asp27) [26] and FucP (Asp46) [35,36]. However, only XylE has a comparable positively charged residue in the vicinity of the putative H^+^-binding site (Arg133 in helix-VI), which casts some doubt on the universality of this mechanism.

**Fig 8:**
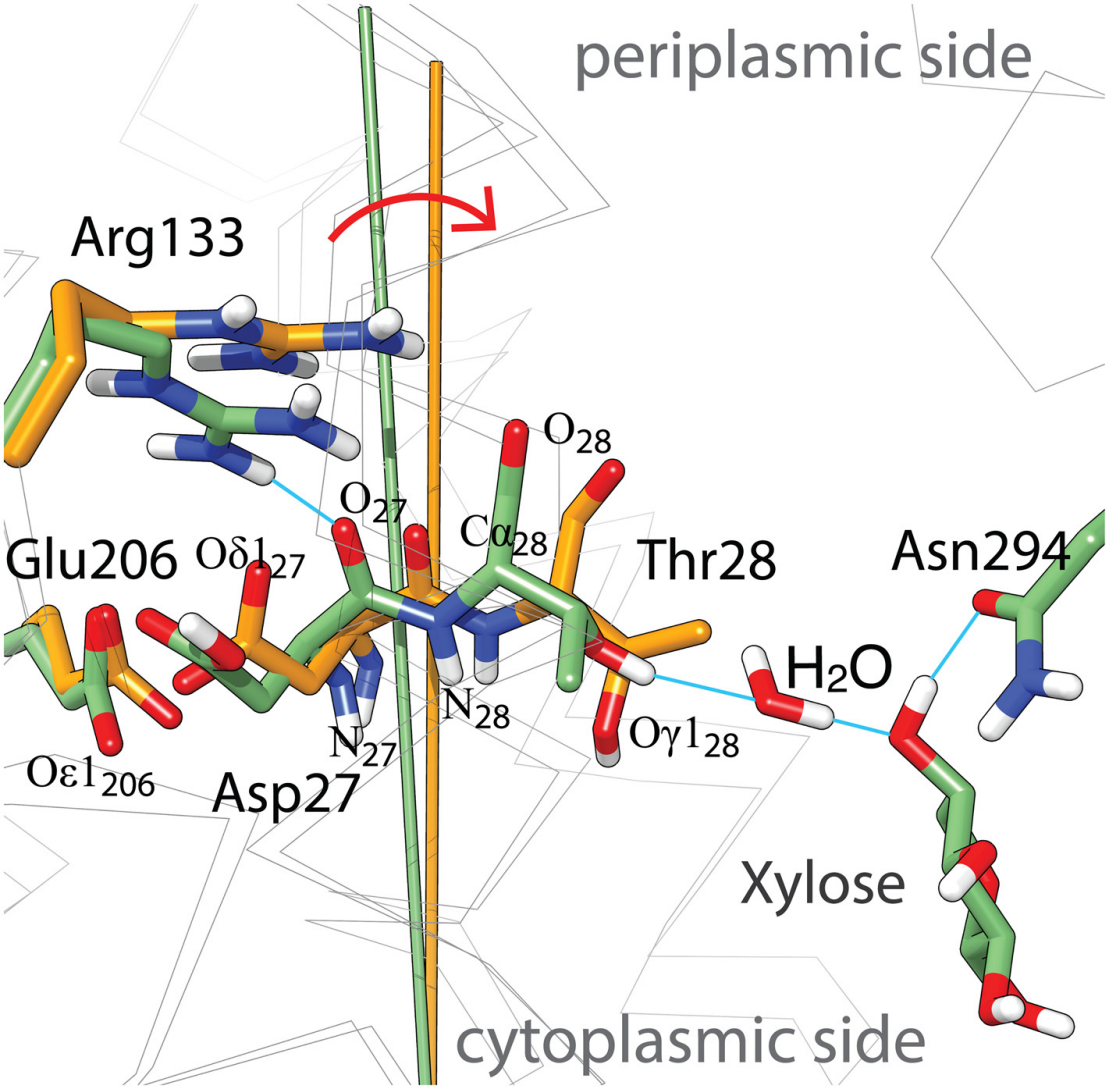
Superposition of xylose-bound (pdb-Id: 4gby, pos. 27, 28, 28, 133, 206, 294 and xylose C atoms shown in green) and substrate-free (pdb-Id: 4qiq, pos.27, 28, 28, 133 and 206 C atoms shown in orange) structure models of XylE focused on the vicinity of Asp27. The stick model shows prominent side chains (heteroatoms are colored red for oxygen, blue for nitrogen and white for hydrogen, blue lines indicate possible H-bounds), remaining Cα-helix traces are indicated as gray wire model,. The batons represent the axis of helix-I in the respective structures and the red arrow illustrates the tilting movement of helix-I. For orientation, the cytoplasmic and periplasmic sides are indicated and few specific atoms are labeled.

However, there is an intriguing alternative mechanism for a pK switch which is illustrated in Fig 8 using the example of XylE. Similar to Glu325 in LacY, the side chain of Asp27 in XylE does not make a direct contact to the glucopyranosyl ring (Fig 8) [20], but an indirect interaction to the C4-OH of xylose is established through Thr28 (helix-I) and H_2_O-606 (Fig 8). Comparison of the xylose-bound with the substrate-free x-ray structure of XylE [15] reveals that the absence of substrate in the binding site leads to a tilting movement of helix-I, away from the helix-VI. The pK of Asp27 is dictated by the non-covalent coupling with another titratable group, the carboxylate of Glu206 (helix-VI), and the proximity to Arg133 (helix-IV). In the substrate-bound structure, the position of the Arg133 guanidinium group indicates an H-bond to the backbone of helix-I, and the pK of Asp27 is expected to be in alkaline region. The widened crossing angle between helix-I and helix-IV upon sugar release likely results in releasing Arg133 from its H-bound position (Fig 8). The positive charge of the guanidinium group would impact the pK of the critical carboxylate at position 27, and the pK is expected to be shifted to the acidic region.

In this view, the transition between low and high pK states is brought about by binding of the substrate rather than by the conformational transition inward-facing to outward-facing, and both, high and low pK states may coexist in both the inward-facing and the outward-facing conformation. This would account for functional symmetry of XylE. Whether this is a realistic scenario for MFS symporters remains to be determined.

## Supporting Information

S1 File Model calculation for an asymmetrical transporter. Based on a 6-state kinetic model pH-dependent activity profiles are calculated and compared for unidirectional and mixed orientation of the symporter in the liposomal membrane.
(DOCX)
