# Supplementary material for: pH Regulation of Electrogenic Sugar/H+ Symport in MFS Sugar Permeases
Source: PLoS One. 2016 May 26;11(5):e0156392. doi: 10.1371/journal.pone.0156392 (PMC4882079; doi:10.1371/journal.pone.0156392)
Supplement: S1 File — Based on a 6-state kinetic model pH-dependent activity profiles are calculated and compared for unidirectional and mixed orientation of the symporter in the liposomal membrane. (DOCX) [file pone.0156392.s001.docx]

**Supporting information S1_File for:**

**pH Regulation of Electrogenic Sugar/H^+^ symport in MFS Sugar Permeases**

Andre Bazzone, M. Gregor Madej, H. Ronald Kaback and Klaus Fendler

**Model calculation for an asymmetrical transporter**

By an “asymmetrical” sugar/H^+^ symporter we will understand in the following a symporter that has a primary pK difference at the out and inside (pK_o_ ≠ pK_i_). This may lead to additional asymmetries of its kinetic parameters as will be discussed below.





***Figure S1:*** *Kinetic model for H^+^/sugar symport. S_x_ = sugar binding/released inside (x=i) or outside (x=o).*

The steady state solution of the kinetic model was calculated in an analogous fashion as for the Na^+^/H^+^ exchanger NhaA([1](#_ENREF_1)). Substrate and H^+^ binding was treated as equilibrium processes described by the respective pK_x_ (H^+^ binding) and K_x_ (sugar binding) at the x = i = inward facing or x = o = outward facing conformation of the symporter. The conformational transitions are described by the rate constants k_x_ and j_x_ (x = f = forward, x = r = reverse). The notations in and out as well as forward and reverse refer to the physiological situation and transport direction.

Asymmetrical pK introduces further asymmetry according to the principle of detailed balance. This could be asymmetrical rate constants (j_f_ ≠ j_r_, k_f_ ≠ k_r_) or asymmetrical sugar binding constants (K_i_ = K_o_) or any combination of these. Since experimental evidence rules out asymmetrical sugar binding for LacY([2](#_ENREF_2)) we choose asymmetrical rate constants for this model calculation.

There is an intuitive explanation for the statement made above that asymmetrical pKs introduce asymmetrical rate constants. Detailed balance reflects the fact, that energy is conserved throughout the transport cycle. Energy is required to lower the pK of the H^+^ binding site when the carrier reorients from an outward-facing to an inward-facing conformation, namely 5.7 kJ/mole per pK unit. This increases the energy of the C_i_H^+^S intermediate relative to the C_o_H^+^S intermediate (Fig S1) thereby slowing down the forward and speeding up the reverse rate constant. Therefore, asymmetrical pKs yield asymmetrical rate constants.

Two cases are considered: (a): only the rate constant of the loaded carrier k_x_ are asymmetrical and (b): the rate constants of the loaded carrier k_x_ and of the unloaded carrier j_x_ are asymmetrical. In the two cases the following equations can be derived from the principle of detailed balance:

Case (a):

$$R=\frac{j_{f}\cdot{10}^{-pK_{i}}\cdot K_{i}}{j_{r}\cdot{10}^{-pK_{0}}\cdot K_{o}}$$

$$k_{f}=\frac{k_{a}}{\sqrt{R}}$$

$$k_{r}=k_{a}\sqrt{R}$$

Case (b):

$$R=\frac{{10}^{-pK_{i}}\cdot K_{i}}{{10}^{-pK_{0}}\cdot K_{o}}$$

$$j_{f}=\frac{k_{b}}{\sqrt{\sqrt{R}}}$$

$$j_{r}=k_{b}\sqrt{\sqrt{R}}$$

$$k_{f}=\frac{k_{b}}{\sqrt{\sqrt{R}}}$$

$$k_{r}=k_{b}\sqrt{\sqrt{R}}$$

The values k_a_=10000 and k_b_=6000 yielded a forward transport rate of ~ 50 s^-1^ in both cases and generated a pH profile as was measured for XylE (see Fig 3 in the main manuscript) together with the remaining kinetic parameters of the table.

The resulting kinetic parameters for case (a) and (b) are given in the table. Note that the conditions were chosen to approximate the concentrations used in our SSM experiments, e.g. pH inside was equal to pH outside the proteoliposomes. Transport is driven by the applied sugar concentration difference. The table shows the case of the right side out (RSO) oriented transporters. Activity of the inside out (ISO) oriented transporters was calculated by reversing the sugar concentrations ([S_o_]=0) and reversing the sign of the activity.

|  | case (a) | case (b) |
| --- | --- | --- |
| K_o_ = K_i_ | 1.7 mM | 1.7 mM |
| pK_o_ | 8.8 | 11.8 |
| pK_i_ | 4.8 | 4.8 |
| k_f_ | 100 s^-1^ | 100 s^-1^ |
| k_r_ | 10^6^ s^-1^ | 3.28^.^ 10^5^ s^-1^ |
| j_f_ | 100 s^-1^ | 100 s^-1^ |
| j_r_ | 100 s^-1^ | 3.28^.^ 10^5^ s^-1^ |
| [S_o_] | 30 mM | 30 mM |
| [S_i_] | 0 | 0 |
| pH_o_ = pH_i_ | variable | variable |

***Table S1:*** *Kinetic parameters for the model calculation shown in Fig S2. The two cases (a) and (b) are explained above.*





***Figure S2:*** *Model calculation for an asymmetrical transporter using the kinetic model in Fig S1. The black red and blue lines show the calculated activity obtained from 100% inside-out oriented transporter, 50% right-side-out and 50% inside-out oriented transporter and 100% right-side-out oriented transporter, respectively, in the proteoliposomes. Rate constants k and j calculated according to the principle of detailed balance (see equations above). Two cases, (a) and (b), are considered as explained in the text.*

The model calculation clearly shows that mixed orientation results in a biphasic acidic inactivation but that the alkaline side of the pH profile is in both cases monophasic. The calculation also shows that characteristically broad pH profiles as experimentally observed are only obtained when the high pK side is outward-oriented as is the case in RSO oriented LacY. This suggests that also FucP and XylE is preferentially RSO oriented in the proteoliposome or, alternatively, as suggested in the manuscript, the pK value asymmetry is a result of the mechanism and not that of a topological difference of in- and outside.

**References**

1. Mager T, Rimon A, Padan E, Fendler K. Transport mechanism and pH regulation of the Na^+^/H^+^ antiporter NhaA from Escherichia coli: An electrophysiological study. J Biol Chem. 2011 May 12;286(26):23570-81. PubMed PMID: 21566125. Epub 2011/05/14. Eng.

2. Guan L, Kaback HR. Binding affinity of lactose permease is not altered by the H+ electrochemical gradient. Proceedings of the National Academy of Sciences of the United States of America. 2004 Aug 17;101(33):12148-52. PubMed PMID: 15304639. Pubmed Central PMCID: 514448.
